# Headache and Other Morbid Cephalic Sensations

**Published:** 1894-09

**Authors:** 


*Headache and other Morbid Cephalic Sensations.* By HARRY CAMPBELL, M.D. Pp. xi., 410. London: H. K. LEWIS. 1894.

Dr. Campbell has brought together in this substantial volume a vast amount of information and suggestion upon this common but ill-understood symptom of headache. Not only does he record his own experience and opinion upon the various points at issue, but, that he has read widely and quoted wisely, the many extracts from other authors, ancient and modern, incorporated in the text and appended in foot-notes, amply prove. It would be superfluous to say that no fault exists in the book—every author and every critic has his own idea of what is most appropriate, but we do venture to express the opinion that it would have been possible to have avoided some diffuseness and to have exercised more compression without the value of the work being diminished. Headache, being a symptom only, could profitably have been discussed from the point of view of symptomatology alone. Almost never are we called upon to treat the symptom directly, but always indirectly through the cause which gives rise to the symptom. Many chapters of the book are excellently done and well repay earnest perusal and study, as they present the best existing account in English of the particular topic treated of; especially in this connection we would mention the chapter on headache caused by disorders of the action of the eye-muscles, external and internal. Altogether, we have to thank Dr. Campbell for a very valuable and suggestive work, which will add largely to his reputation as a scientific clinician, and if he were to bring out an abstract of the text arranged in a synoptical form—devoid alike of prolixity and conflicting opinion—it would fill another distinct gap in our medical literature.

